# Low expression of TGF‐β2 and matrilin2 in human aqueous humour with acute primary angle closure

**DOI:** 10.1111/jcmm.18111

**Published:** 2024-01-18

**Authors:** Liming Wang, Zhao Xu, Yaru Hong, Yan Liu, Xiaomin Zhang, Qiang Feng, Dandan Zhang, Kexi Chen, Guli Humaer Yiming, Xiaorong Li, Aihua Liu, Lijie Dong

**Affiliations:** ^1^ Tianjin Key Laboratory of Retinal Functions and Diseases, Tianjin Branch of National Clinical Research Center for Ocular Disease Eye Institute and School of Optometry, Tianjin Medical University Eye Hospital Tianjin China; ^2^ Ophthalmology Department of People's Hospital of Hotan District Xinjiang China

**Keywords:** acute primary angle closure, aqueous humour, ELISA, glaucoma, matrilin2, proteome, TGF‐β2

## Abstract

Primary angle‐closure glaucoma (PACG) is the leading cause of irreversible blindness in the world. Angle closure induced by pupil block and secondary iris synechia is the fundamental pathology of the PACG. The molecular mechanisms of angle closure have not yet been clearly illustrated. This study was designed to investigate the protein difference in the aqueous humour and explore new biomarker of the PACG. Aqueous humour (AH) was collected from patients with acute primary angle closure (APAC) and cataract (*n* = 10 in APAC group) and patients with cataract only (*n* = 10 in control group). Samples were pooled and measured using label‐free proteome technology. Then, the differentially expressed proteins (DEPs) were verified by ELISA using independent AH samples (*n* = 20 each group). More than 400 proteins were revealed in both groups through proteomics. Comparing the two groups, there were 91DEPs. These proteins participate in biological activities such as inflammation, fibrosis, nerve growth and degeneration and metabolism. We found that the expression of transforming growth factor‐β2 and matrilin2 was downregulated in the APAC group. The two proteins are related to inflammation and extracellular matrix formation, which might be involved in angle closure. This study characterized DEPs in AH of the APAC and found a downregulated protein matrilin2.

## 1 INTRODUCTION

Acute primary angle closure (APAC) is an ophthalmic emergency comprising complaints of sudden headache, nausea or vomiting, eye pain and redness and blurry vision. Eyes with APAC usually have a narrow anterior chamber angle and forward bowing of the peripheral iris. This abnormal anatomy obstructs aqueous humour (AH) outflow and results in a sudden elevation in intraocular pressure (IOP) and acute optic nerve damage, manifesting as abruptly reduced vision, afferent pupillary defects, optic disc oedema and disc haemorrhages, which are similar to those with ischemia injury.^1^, ^2^, ^3^, ^4^ Without timely treatment, it can lead to iris synechia, angle dysfunction, higher IOP and irreversible optic nerve damage. Optic nerve head pallor, vision loss and visual field deficits, which are the characteristic symptoms of advanced glaucoma.^5^, ^6^, ^7^ Currently, the management of angle‐closure glaucoma includes laser periphery iridotomy, lens extraction and pharmaceutical treatment.^8^, ^9^, ^10^ However, these treatments are insufficient for the protection of optic nerves. Multiple studies have been conducted on neuroprotection,^11^, ^12^, ^13^ but research on the micro‐environment of the anterior chamber is limited.

The AH is a fluid in the eyes that maintains IOP, regulates the metabolism of anterior segment tissues and reflects ophthalmic pathophysiology.^14^ AH extraction is minimally invasive and frequently indicated for the diagnosis and treatment of eye diseases.^15^, ^16^ Previous studies have analysed various glaucoma biomarkers with different methods, which include inflammation, oxidative stress, neurotrophy factors and some antibodies.^17^, ^18^, ^19^ However, no clear method has yet been developed. Mass spectrometry‐based proteomic and bioinformatic methods have enabled the comparison of proteins in a variety of biological samples with a high level of resolution and accuracy.^20^ The application of this technology to different diseases has improved our understanding of disease mechanisms^21^, ^22^, ^23^ and highlighted new disease biomarkers and therapeutic methods.^24^, ^25^, ^26^

In this study, we initially hypothesized that angle closure is related to AH change in primary angle‐closure glaucoma (PACG) and some proteins in AH could reflect the anterior chamber angle before neuropathy. The aim of this study was to analyse the characteristics of differentially expressed proteins (DEPs) based on the AH proteomes of APAC patients and to discover valuable information for treatment in the early stage of PACG.

## 2 MATERIALS AND METHODS

### 2.1 Patients

This study was approved by the Tianjin Medical University Eye Hospital Ethics Committee [2016KY(L)‐26] and complied with the standards of the Declaration of Helsinki for human experiments. Patients provided signed informed consent. Sixty patients were recruited for the study, including 30 patients with APAC and cataracts (APAC group) and 30 patients with cataracts (control group). Samples from 10 patients in each group were used for the proteome experiment, and which from 20 patients in each group were used for the enzyme‐linked immunosorbent assay (ELISA). All patients underwent a series of ophthalmic examinations by glaucoma specialists, including IOP, visual acuity, slit lamp, gonioscopy, B‐mode ultrasound, ultrasound biomicroscopy, optical coherence tomography, fundus photography and Humphrey visual field examination. Inclusion criteria for APAC and cataract patients were as follows: IOP > 30 mmHg (1 mmHg = 0.133 kPa), as well as symptoms including conjunctival congestion, corneal oedema, dilated pupil and shallow anterior chamber observed through the slit lamp. The angle closure (>180°) was confirmed through angioscopy or ultrasound biomicroscopy.^10^ Patients with APAC also had a cataract diagnosis based on the slit lamp. The diagnostic criteria for cataracts were opacity of the lens and gradual loss of vision. The control subjects had no history of glaucoma. Premedication: patients with APAC took eye drops including decreasing IOP and antibiotic, patients with cataract only dropped antibiotic. Medicines decreasing IOP include carteolo hydrochloride, brinzolamide and brimonidine tartrate. Antibiotic is levofloxacin. Patients were excluded from the study if they had other ophthalmic diseases, such as trauma, inflammation or secondary glaucoma, or they have been were received eye surgery. AH samples (50–100 μL) were collected through a 1‐mL syringe during surgery. The samples in a sterile tube were placed on ice, then centrifuged at 4°C (2000 rpm, ~400 *g*, ×5 min) and finally stored in the freezer (−80°C).

### 2.2 Total protein estimation using Bradford's method

The Bradford method has been applied for protein quantification for many years.^27^ Total protein concentrations in AH samples were determined using Bradford's Assay Kit (Thermo Fisher Scientific, USA). Taking 10 μL of diluted sample or different concentration BSA (bovine serum albumin) solution into a 96‐well plate, mixed with 300 μL of protein quantification dye and then reacted for 20 min in the dark. The absorbance under 595 nm was measured by a microplate reader. The standard curve was generated according to the absorbance value of different concentration BSA. According to the standard curve formula, the protein concentration of each sample could be calculated.

### 2.3 Proteolysis

The method of proteolysis was based on sodium dodecyl sulphate polyacrylamide gel electrophoresis as performed previously.^26^ The SDS‐PAGE was performed with 5 μg of protein per sample; the gel was stained and then destained until the background was clear. The gel was cut into pieces of approximately 1 mm^3^, placed in a 1.5 mL Eppendorf tube and washed with 200 μL of ultra‐pure water twice for 10 min. Coomassie brilliant blue destaining buffer was added [50 mM NH_4_HCO_3_ and acetonitrile (ACN) (1:1)], and the gel was destained for 15 min before washing with ultra‐pure water. This was repeated three times until detaining completely. After that, 100 μL of ACN was added to dehydrate the gel particles until they were white. Subsequently, 200 μL of 10 mM C_4_H_10_O_2_S_2_ (DTT) was added to a warm water bath for 60 min before adding 100 μL of ACN to dehydrate gel particles. Then, 200 μL of 55 mM indole‐3‐acetic acid was added before placing it in the dark for 30 min and adding 100 μL of ACN to dehydrate the gel particles. The following solutions were used to carry out the suspension: ultra‐pure water (once), ACN (once), ultra‐pure water (once) and ACN (once), each for 10 min. Then, 100 μL of 0.01 μg/μL trypsin was added to each tube, allowing the enzyme solution and gel particles to completely contact for 30 min at 4°C; this was stopped when the trypsin was absorbed by the gel, and 100 μL of 25 mM NH_4_HCO_3_ was then added. The mixture was incubated at 37°C overnight. The next day, the supernatant was collected and transferred into a clean Eppendorf tube. After adding 5% CF_3_COOH (TFA) and 95% double distilled water (ddH_2_O) extraction buffer to the pellets for 1 h, the collected supernatants were transferred into the corresponding Eppendorf tube. The pellets were treated with extraction buffer (2.5% TFA, 50% ACN, 47.5% ddH_2_O) for 1 h, and the supernatant was placed in the previous Eppendorf tubes. Finally, the peptides were vacuum dried and stored at −20°C.

### 2.4 Q‐exactive for proteomic and data analysis

Instruments used in the experiment: Mass spectrum (Thermo, Q‐Exactive), high‐performance liquid chromatograph [Thermo Scientific EASY‐nLC 1000 System (Nano HPLC)], Centrifuge (Thermo, PICO17) and Vortex Oscillator (Haimen Kylin‐Bell Lab Instruments Co. Ltd., QL‐901). Mass spectrometry parameter settings: spray voltage: 210 V; capillary temperature: 250°C; Ion Source: EASY‐Spray source; Experimental procedures: The fractions obtained through high‐pH reverse‐phase separation were eluted with 20 μL of solution (2% methanol and 0.1% formic acid) and centrifuged at 11,269 *g* for 10 min, and 10 μL of supernatant was loaded according to the sandwich method. The flow rate of the loading pump was set to 350 nL/min for 15 min, and the flow rate of separation was 350 nL/min. The separation gradients in phase B [time (min)/percentage (%)] were as follows: 0/4, 5/15, 40/25, 65/35, 70/95, 82/95, 85/4 and 90/4. Raw data were analysed using Max Quant software (version 1.6.2.0.). The biological information of these DEPs was obtained based on some bioinformation data websites or database, such as https://www.uniprot.org, http://www.Genome.jp/kegg and the STRING database.

### 2.5 Verification of DEPs by performing ELISA

AH samples from patients with cataracts (*n* = 20) and patients with APAC and cataracts (*n* = 20) were used for verification with ELISA kits [TGF‐β2 (ml061149), CDH4 (ml370346), ANXA1 (ml038038), MATN2 (ml761904)] (Mlbio, Shanghai). The procedure followed the manufacturer's introductions. The calculation formula of the target protein is as follows: Target protein = target protein concentration/total protein concentration × sample volume × dilution factor.

### 2.6 Statistical analysis

MaxQuant significances A was used to assess the differential proteins in the proteome experiment. Differences were considered statistically significant when the fold‐change was greater than 1.5 and the *p*‐value was less than 0.05. Subject information and ELISA results were tested by performing an unpaired *t*‐test followed by the Mann–Whitney *U* test using GraphPad® Prism 7 software. Data were expressed as the mean ± SD, and *p* < 0.05 was regarded as statistically significant.

## 3 RESULTS

In this study, samples of AH were collected from patients with APAC and cataract (APAC group) and cataract only (control group). Through proteome method, we obtained and analysed the DEPs in the APAC group (*n* = 10, pooled) compared with the control group (*n* = 10, pooled). The result demonstrates that these proteins are involved in inflammation, metabolism, nerve, adhesion and fibrosis, as illustrated by Gene Ontology (GO), Kyoto Encyclopedia of Genes and Genomes (KEGG) and protein–protein interaction (PPI). Based on the biological function, we chose four proteins and verified them in the APAC and control group (*n* = 20/group, independent sample) by ELISA. Finally, we agree that matrilin2 (MATN2)/transforming growth factor‐β2 (TGF‐β2) expression was downregulated in the APAC group based on both methods.

### 3.1 Subjects

**FIGURE 1:**
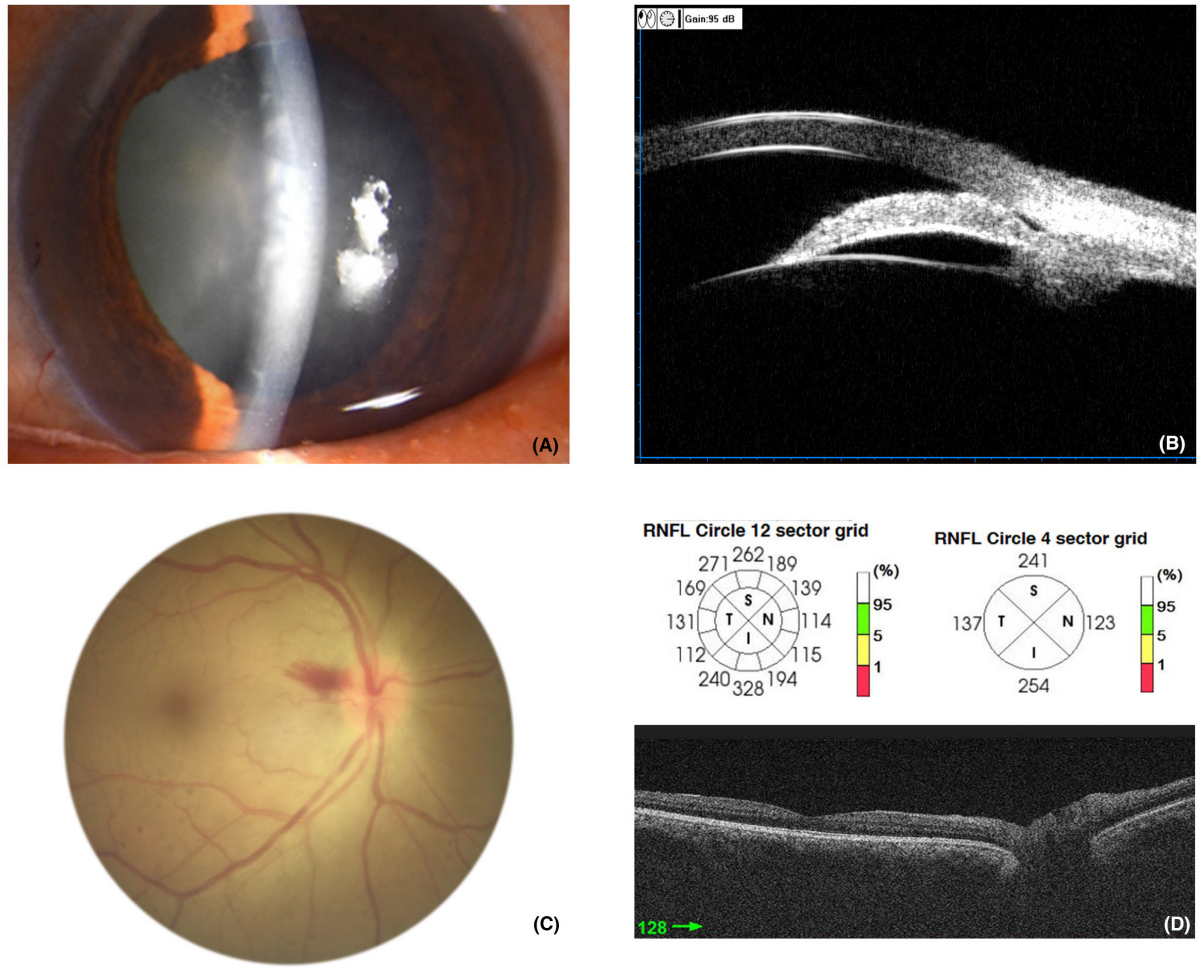
Examination of the acute primary angle‐closure (APAC) patients. Corneal oedema, shallow anterior chamber, dilated pupil and local iris atrophy were observed via slit‐lamp microscopy in the eye of APAC patient (A). The closed angle and bulging iris are shown based on ultrasound biomicroscopy in the eye of the APAC patient (B). The optic disc had clear boundaries, close‐to‐normal colour and a piece of flame‐like bleeding beside the optic disc, representing an attack sign in the fundus image (C). The thickness of the nerve fibre layer was within the normal range in optical coherence tomography (D).

According to the guidelines of the fifth European Glaucoma Society and American Academy of Ophthalmology, patients in APAC group had high IOP (>21 mmHg), closed anterior chamber angle and opacified lens, but still in the normal thickness of the retinal nerve fibre layer (Figure 1). Patients in the control group only manifest opacified lens. Table 1 shows the subjects' information in the proteome experiment. The average age has no obviously different between the APAC group (*n =* 10) and the control group (*n* = 10), which are 68.00 ± 1.86 and 71.70 ± 2.51 years, respectively (*p* = 0.25). There is no significant difference in the sex distribution, cup/disc ratio and visual acuity between the groups (*p* > 0.05); however, there is statistical difference in the mean IOP, anterior chamber depth, axial length and corneal thickness when comparing the two groups (*p* < 0.05). Table 2 shows the subjects' information in the ELISA test. The mean age in the APAC (*n* = 20) and control (*n* = 20) group show 67.70 ± 1.60 and 72.20 ± 1.63 years, which has no obvious difference (*p* > 0.05) and there is no significant difference in the sex distribution and visual acuity (*p* > 0.05), however, there is significant difference in the mean cup/disc ratio, IOP, axial length, corneal thickness and anterior chamber depth (*p* < 0.05).

**TABLE 1:** Demographic and clinical characteristics of cataract and APAC subjects for the proteome.

Characteristics	APAC combined cataract	Cataract	*p*‐Value	Statistical difference
Subjects, *n*	10	10		
Male/female	3/7	2/8	0.0698	ns
Age, year (Mean ± SD)	68.00 ± 1.86	71.70 ± 2.51	0.2516	ns
Cup/disc ratio (Mean ± SD)	0.38 ± 0.01	0.35 ± 0.02	0.1769	ns
IOP (Mean ± SD), mmHg	37.98 ± 2.10	14.34 ± 1.58	0.0006	***
Axial length (Mean ± SD), mm	22.50 ± 0.27	23.86 ± 0.25	0.0015	**
Corneal thickness (Mean ± SD), μm	575.70 ± 14.03	521.00 ± 9.11	0.0043	**
Anterior chamber depth (Mean ± SD), mm	2.10 ± 0.06	3.27 ± 0.15	0.0001	***
VA (Mean ± SD)	0.33 ± 0.12	0.31 ± 0.07	0.9174	ns
Other eye diseases history	—	—	—	—

*Note*: Statistical analysis: Nonparametric *t*‐test (* represents *p* < 0.05, ** represents *p* < 0.01, *** represents *p* < 0.001, ns represents no significant difference).

Abbreviations: APAC, acute primary angle closure; IOP, intraocular pressure; SD, standard deviation; VA, visual acuity.

**TABLE 2:** Demographic and clinical characteristics of cataract and APAC subjects for the ELISA.

Characteristics	APAC combined cataract	Cataract	*p*‐Value	Statistical difference
Subjects, *n*	20	20		
Male/female	3/17	8/12	0.0802	ns
Age, year (Mean ± SD)	67.70 ± 1.60	72.20 ± 1.63	0.0564	ns
Cup/disc ratio (Mean ± SD)	0.58 ± 0.03	0.45 ± 0.04	0.0097	**
IOP (Mean ± SD), mmHg	34.72 ± 3.76	13.95 ± 0.53	0.0001	***
Axial length (Mean ± SD), mm	22.02 ± 0.19	23.55 ± 0.24	0.0001	***
Corneal thickness (Mean ± SD), μm	578.40 ± 13.93	534.70 ± 6.53	0.0072	**
Anterior chamber depth (Mean ± SD), mm	2.11 ± 0.05	3.10 ± 0.10	0.0001	***
VA (Mean ± SD)	0.25 ± 0.05	0.19 ± 0.04	0.3951	ns
Other eye diseases history	—	—	—	

*Note*: Statistical analysis: Nonparametric *t*‐test (* represents *p* < 0.05, ** represents *p* < 0.01, *** represents *p* < 0.001, ns represents no significant difference).

Abbreviations: IOP, intraocular pressure; SD, standard deviation; VA, visual acuity.

### 3.2 Data acquisition

**FIGURE 2:**
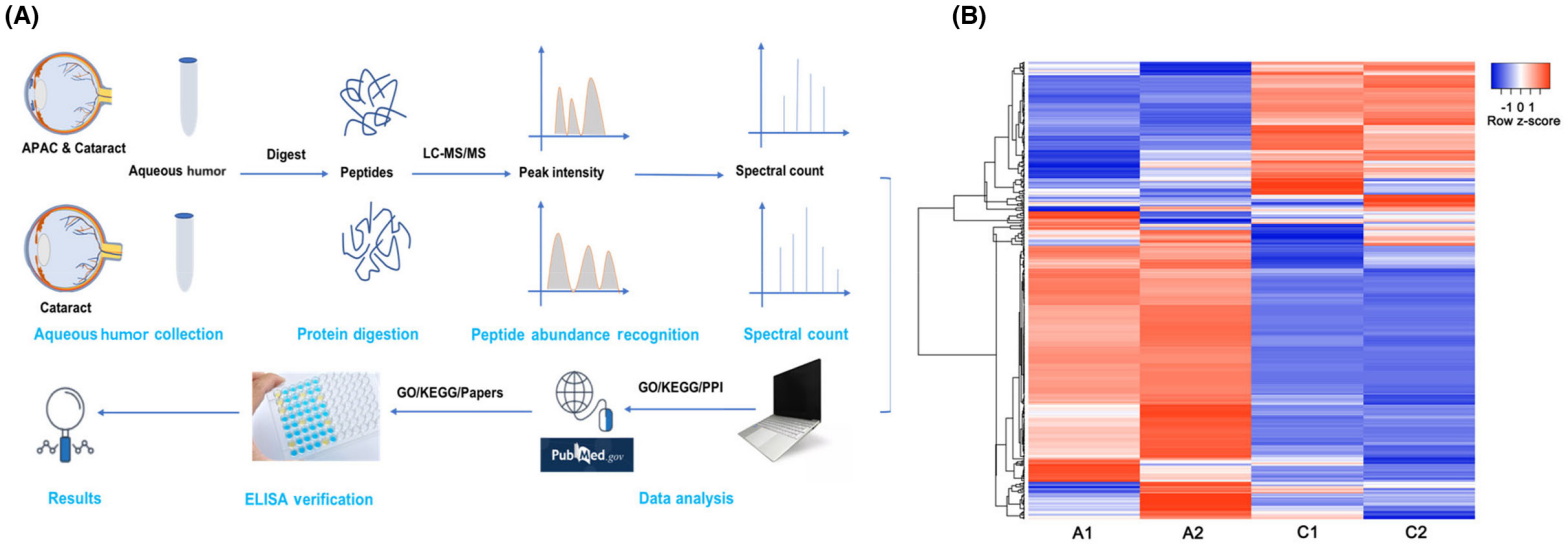
Workflow of proteomic technology and heatmap of the aqueous humour proteins in acute primary angle‐closure (APAC) group and controls. The workflow shows aqueous humour (AH) collection, protein digestion, peptide abundance recognition, spectral count with label‐free proteomic technology, data analysis and enzyme‐linked immunosorbent assay (ELISA) verification (A). The heatmap shows protein expression in the AH of the APAC group (A1, A2) and controls (C1, C2). Red represents upregulated proteins and blue represents downregulated proteins (B).

The workflow of the label‐free proteome test includes four key steps as follows: protein digestion, peptide abundance recognition, spectral counting and data analysis (Figure 2A). Each group was tested twice (A1A2 and C1C2). In the heatmap, protein levels showed noticeable differences between the groups. The colours present upregulated and downregulated proteins, marked with red and blue, respectively (Figure 2B).

### 3.3 Data analysis: GO, KEGG, PPI

**FIGURE 3:**
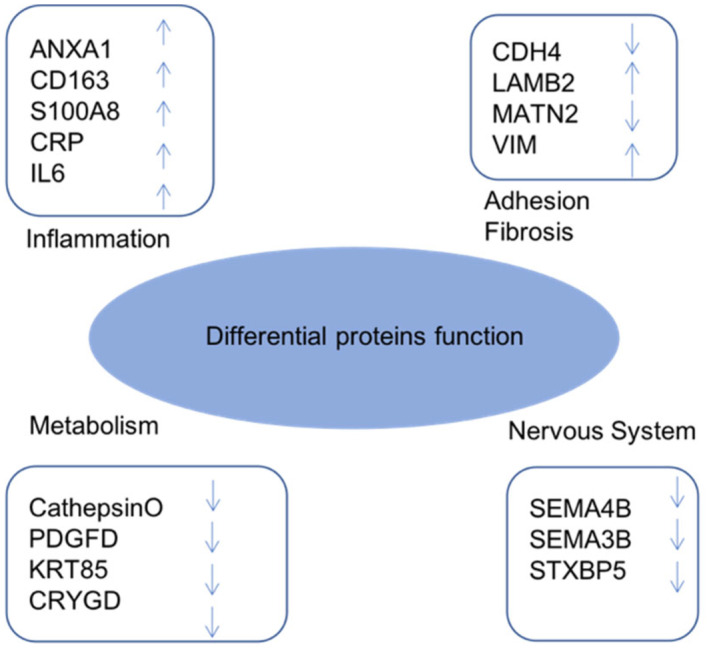
Classification of significantly DEPs. According to their function, the DEPs are determined to be mainly involved in inflammation, metabolism, the nerve system, adhesion and fibrosis. Arrows represent protein increase or decrease in the APAC aqueous humour (AH) in comparison to control group.

Approximately 400 proteins were identified through proteome technology. Table 3 lists 91 significant DEPs (fold‐change >1.5 and *p* < 0.05). There were 50 upregulated and 41 downregulated proteins in the APAC group compared with levels in the control group. The interesting proteins are labelled with yellow in Table 3, which are related to inflammation, metabolism, cellular adhesion and nerve growth and degeneration in terms of biological function (Figure 3).

**TABLE 3:** All differential proteins between APAC and cataract based on proteome.

Uniprot ID	Gene name		FC.A/C	*p* Value. A/C	Sig. (A/C)
CRP_HUMAN	CRP	C‐reactive protein	479	0.0000	1
PZP_HUMAN	PZP	Pregnancy zone protein	118	0.0013	1
STC1_HUMAN	STC1	Stanniocalcin−1	105	0.0017	1
CRYAA_HUMAN	CRYAA	Alpha‐crystallin A chain	98	0.0020	1
LAMC1_HUMAN	LAMC1	Laminin subunit gamma−1	95	0.0021	1
LBP_HUMAN	LBP	Lipopolysaccharide‐binding protein	80	0.0031	1
F5GZZ9_HUMAN	CD163	Scavenger receptor cysteine‐rich type 1 protein M130	69	0.0042	1
E5RFN6_HUMAN	ABCA10	ATP‐binding cassette sub‐family A member 10	67	0.0044	1
E9PHM6_HUMAN	DST	Dystonin	67	0.0045	1
CBPN_HUMAN	CPN1	Carboxypeptidase N catalytic chain	67	0.0045	1
CH3L2_HUMAN	CHI3L2	Chitinase‐3‐like protein 2	63	0.0051	1
B5MCZ3_HUMAN	IL6	Interleukin‐6	56	0.0064	1
HGFL_HUMAN	MST1	Hepatocyte growth factor‐like protein	54	0.0070	1
SPB3_HUMAN	SERPINB3	Serpin B3	50	0.0081	1
HBG1_HUMAN	HBG1	Haemoglobin subunit gamma−1	48	0.0089	1
HABP2_HUMAN	HABP2	Hyaluronan‐binding protein 2	47	0.0090	1
TALDO_HUMAN	TALDO1	Transaldolase	44	0.0103	1
DSC2_HUMAN	DSC2	Desmocllin‐2	39	0.0129	1
ANXA1_HUMAN	ANXA1	Annexin A1	39	0.0131	1
NID1_HUMAN	NID1	Nidogen‐1/laminin	37	0.0140	1
S10A4_HUMAN	S100A4	Protein S100‐A4	37	0.0145	1
CAPG_HUMAN	CAPG	Macrophage‐capping protein	35	0.0160	1
TKT_HUMAN	TKT	Transketolase	34	0.0166	1
LDHB_HUMAN	LDHB	L‐lactate dehydrogenase B chain	32	0.0187	1
GNPTG_HUMAN	GNPTG	N‐acetylglucosamine−1‐phosphotransferase subunit gamma	31	0.0196	1
NRX1A_HUMAN	NRXN1	Neurexin‐1	30	0.0204	1
D6REY1_HUMAN	CHIT1	Chitotriosidase‐1	30	0.0210	1
KCRB_HUMAN	CKB	Creatine kinase B‐type	29	0.0219	1
IGHM_HUMAN	IGHM	Immunoglobulin heavy constant mu	28	0.0234	1
B0YJC4_HUMAN	VIM	Vimentin	27	0.0243	1
SNED1_HUMAN	SNED1	Sushi, nidogen and EGF‐like domain‐containing protein 1	26	0.0256	1
GDN_HUMAN	SERPINE2	Glia‐derived nexin	26	0.0268	1
H3BTN5_HUMAN	PKM	Pyruvate kinase (Fragment)	26	0.0269	1
LAMB2_HUMAN	LAMB2	Laminin subunit beta‐2	25	0.0272	1
A0A087WVQ9_HUMAN	EEF1A1	Elongation factor 1‐alpha 1	24	0.0290	1
HBD_HUMAN	HBD	Haemoglobin subunit delta	23	0.0309	1
NID2_HUMAN	NID2	Nidogen‐2	23	0.0319	1
COTL1_HUMAN	COTL1	Coactosin‐like protein	22	0.0339	1
F5H6I0_HUMAN	B2M	Beta‐2‐microglobulin	22	0.0342	1
CYTB_HUMAN	CSTB	Cystatin‐B	22	0.0343	1
CD5L_HUMAN	CD5L	CD5 antigen‐like	21	0.0354	1
PCSK1_HUMAN	PCSK1N	ProSAAS	21	0.0357	1
S10A8_HUMAN	S100A8	Protein S100‐A8	21	0.0360	1
HPTR_HUMAN	HPR	Haptoglobin‐related protein	21	0.0361	1
ITIH3_HUMAN	ITIH3	Inter‐alpha‐trypsin inhibitor heavy chain H3	21	0.0380	1
C9JP14_HUMAN	ADH7	All‐trans‐retinol dehydrogenase [NAD(+)] ADH7	19	0.0415	1
CPN2_HUMAN	CPN2	Carboxypeptidase N subunit 2	19	0.0424	1
DESP_HUMAN	DSP	Desmoplakin	19	0.0445	1
J3QRJ3_HUMAN	THY1	Thy‐1 membrane glycoprotein	18	0.0447	1
E9PLJ3_HUMAN	CFL1	Cofilin‐1	18	0.0460	1
VASN_HUMAN	VASN	Vasorin	4	0.0489	−1
MATN2_HUMAN	MATN2	Matrilin‐2	4	0.0432	−1
CRBB2_HUMAN	CRYBB2	Beta‐crystallin B2	4	0.0428	−1
ANGI_HUMAN	ANG	Angiogenin	4	0.0419	−1
CRGD_HUMAN	CRYGD	Gamma‐crystallin D	4	0.0412	−1
NEUS_HUMAN	SERPINI1	Neuroserpin	4	0.0397	−1
CPVL_HUMAN	CPVL	Probable serine carboxypeptidase	4	0.0376	−1
CRBA4_HUMAN	CRYBA4	Beta‐crystallin A4	4	0.0375	−1
F5GZ08_HUMAN	APLP1	Amyloid‐like protein 1	4	0.0364	−1
CADH4_HUMAN	CDH4	Cadherin‐4	4	0.0348	−1
COL12_HUMAN	COLEC12	Collectin−12	5	0.0318	−1
PDGFD_HUMAN	PDGFD	Platelet‐derived growth factor D	5	0.0284	−1
NAGAB_HUMAN	NAGA	Alpha‐N‐acetylgalactosaminidase	5	0.0283	−1
G3XAP6_HUMAN	COMP	Cartilage oligomeric matrix protein	5	0.0273	−1
HV205_HUMAN	IGHV2‐5	Immunoglobulin heavy variable 2–5	5	0.0271	−1
A0A0G2JPR0_HUMAN	C4A	Complement C4‐A	5	0.0250	−1
TGFB2_HUMAN	TGF‐β2	Transforming growth factor‐β2 proprotein	5	0.0233	−1
DPP2_HUMAN	DPP7	Dipeptidyl peptidase 2	6	0.0206	−1
HV118_HUMAN	IGHV1‐18	Immunoglobulin heavy variable 1–18	6	0.0194	−1
CATO_HUMAN	CTSO	Cathepsin O	6	0.0187	−1
I3L1J2_HUMAN	CDH5	Cadherin‐5	6	0.0162	−1
PI16_HUMAN	PI16	Peptidase inhibitor 16	6	0.0160	−1
SEM3B_HUMAN	SEMA3B	Semaphorin‐3B	6	0.0159	−1
CBPQ_HUMAN	CPQ	Carboxypeptidase Q	7	0.0149	−1
C9J973_HUMAN	NHLRC3	NHL repeat‐containing protein 3	7	0.0143	−1
HV226_HUMAN	IGHV2‐26	Immunoglobulin heavy variable 2‐26	7	0.0137	−1
HV169_HUMAN	IGHV1‐69	Immunoglobulin heavy variable 1‐69	7	0.0130	−1
CRBS_HUMAN	CRYGS	Gamma‐crystallin S	8	0.0090	−1
ANAG_HUMAN	NAGLU	Alpha‐N‐acetylglucosaminidase	9	0.0064	−1
B3KTY4_HUMAN	SLITRK2	SLIT and NTRK‐like family, member 2, isoform CRA_b	9	0.0062	−1
C9JPG5_HUMAN	SEMA3F	Semaphorin‐3F	10	0.0052	−1
B7WNR0_HUMAN	ALB	Serum albumin	11	0.0043	−1
IDS_HUMAN	IDS	Iduronate 2‐sulfatase	13	0.0024	−1
SEM4B_HUMAN	SEMA4B	Semaphorin‐4B	19	0.0009	−1
DIAC_HUMAN	CTBS	Di‐N‐acetylchitobiase	21	0.0006	−1
KRT82_HUMAN	KRT82	Keratin, type II cuticular Hb2	21	0.0006	−1
EPDR1_HUMAN	EPDR1	Mammalian ependymin‐related protein 1	39	0.0001	−1
NTRI_HUMAN	NTM	Neurotrimin	44	0.0001	−1
KRT85_HUMAN	KRT85	Keratin, type II cuticular Hb5	49	0.0000	−1
H0Y332_HUMAN	STXBP5	Syntaxin‐binding protein 5	244	0.0000	−1
X6R5A3_HUMAN	TDP2	TRAF and TNF receptor associated protein, isoform CRA_b	267	0.0000	−1

**FIGURE 4:**
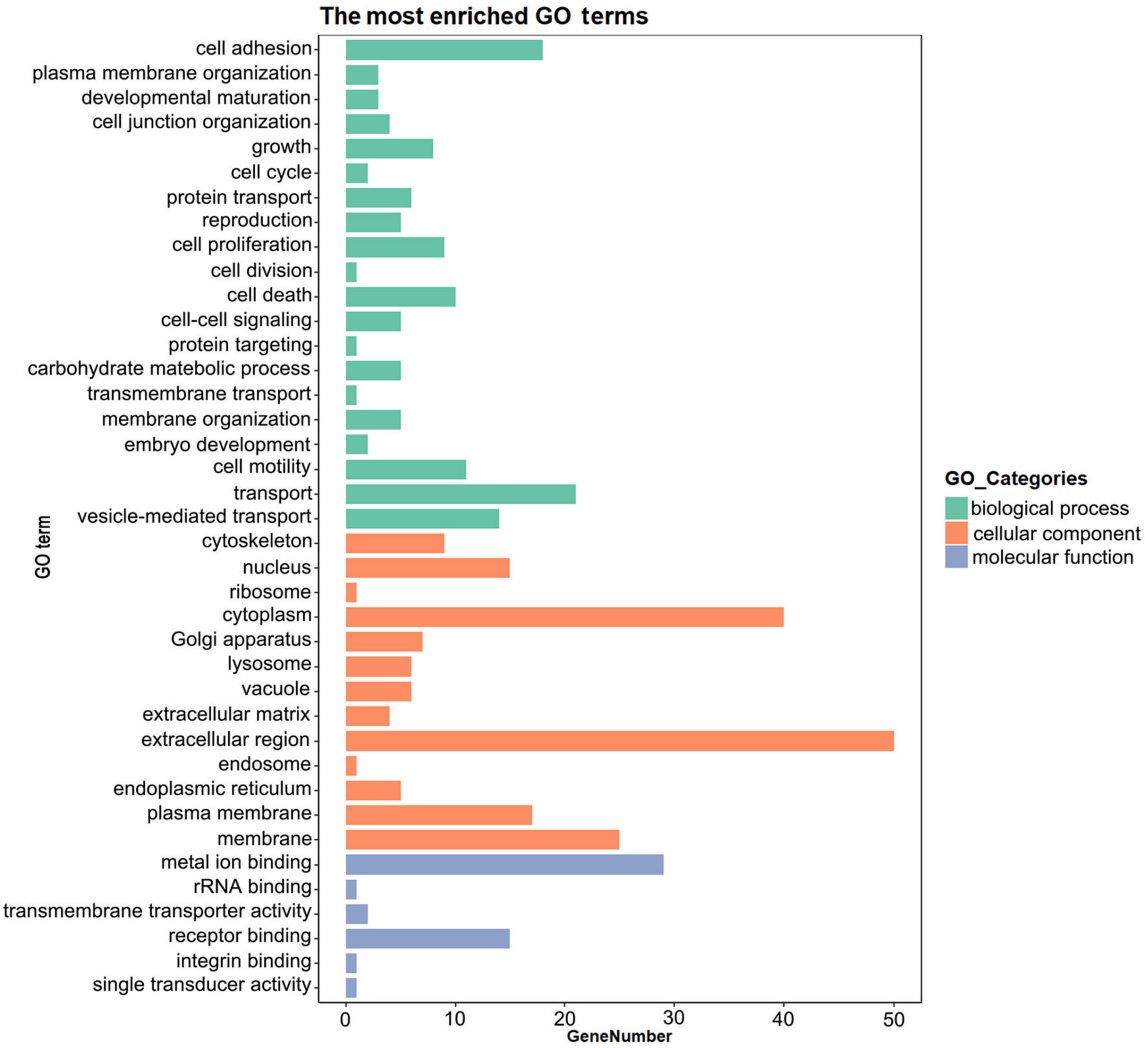
Gene Ontology (GO) analysis of genes of DEPs in the acute primary angle‐closure (APAC) group compared with the cataract group. Illustrated in the histogram, GO analysis includes the biological processes (BPs: green), cellular components (CCs: orange) and molecular functions (MFs: blue). The protein‐encoding genes are mainly enriched in transport, cell motility, cell adhesion, cell proliferation and cell death processes. They are determined to be located in the extracellular region, membrane, cytoplasm, nucleus and cytoskeleton and to participate in metal ion binding, receptor binding and transmembrane transporter activity.

GO analysis shows functions of differentially expressed protein involving biological process, molecular function and cellular component (Figure 4). With respect to biological function, these proteins are determined to be involved in transport, cell adhesion, cell proliferation, cell motility and cell death. In regard to molecular function, they are found to participate in receptor binding, metal ion binding and transmembrane activity. In cellular components, the proteins are enriched in the extracellular matrix, cytoplasm, nucleus and cytoskeleton regions.

**FIGURE 5:**
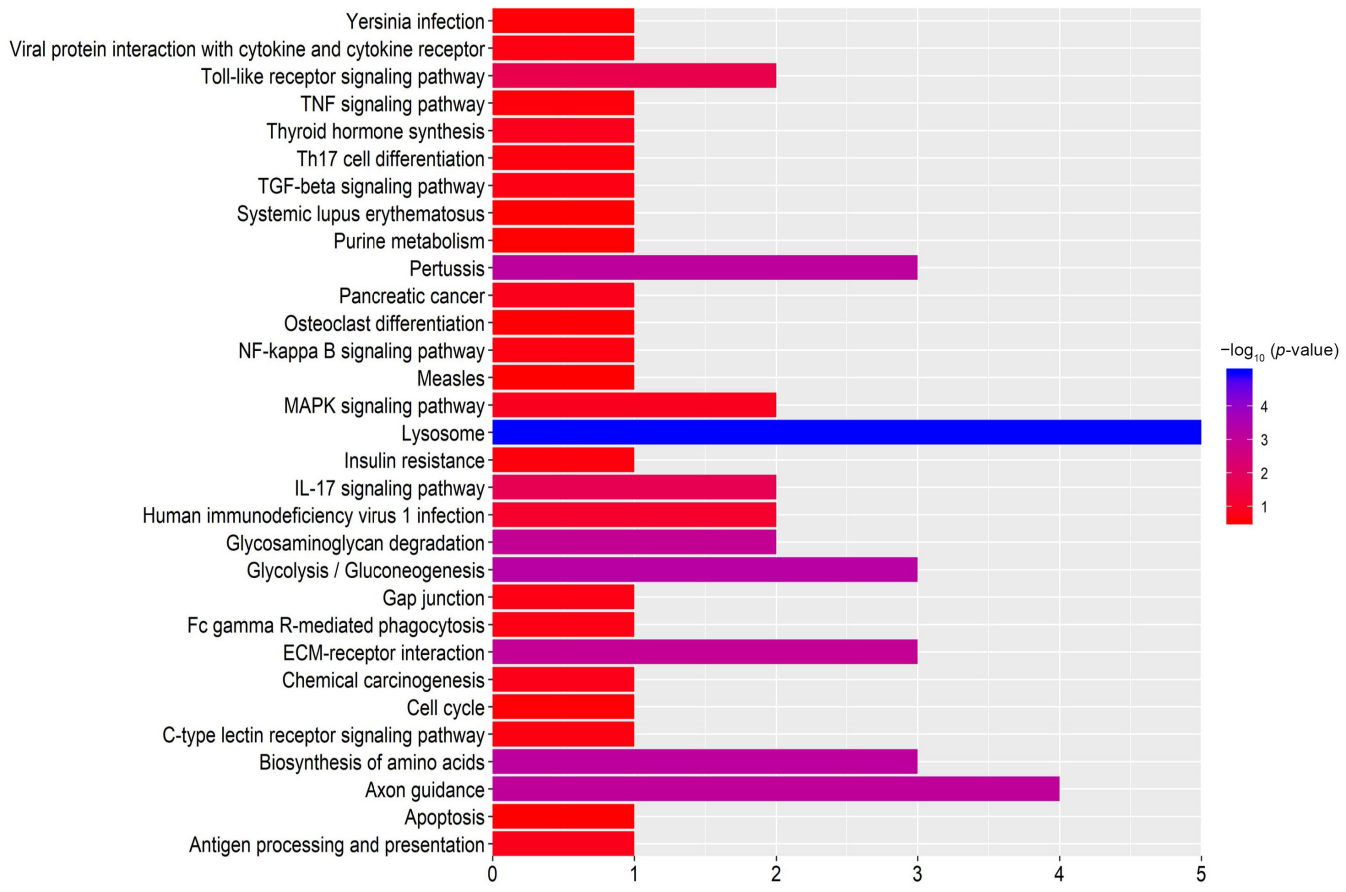
Analysis of the signalling pathways of significantly DEPs in APAC group compared with control by the Kyoto Encyclopedia of Genes and Genomes (KEGG). Based on the *p*‐value, the DEPs were mainly enriched in the lysosome, axon, glycolysis/gluconeogenesis, extracellular matrix (ECM)–receptor interaction and biosynthesis of amino acids signalling pathways. Those proteins participate in some inflammation signalling pathways, such as the toll‐like receptor signalling pathway, MAPK signalling pathway and IL‐17 signalling pathway.

KEGG analysis shows that the DEPs are enriched in multiple pathways, such as the lysosome, axon, glycolysis/gluconeogenesis, extracellular matrix (ECM)–receptor interaction and biosynthesis of amino acids signalling pathways. Additionally, some proteins are also determined to be involved in inflammation pathways, such as the toll‐like receptor, mitogen‐ activated protein kinase (MAPK) and interleukin‐17 (IL‐17) signalling pathways (Figure 5).

**FIGURE 6:**
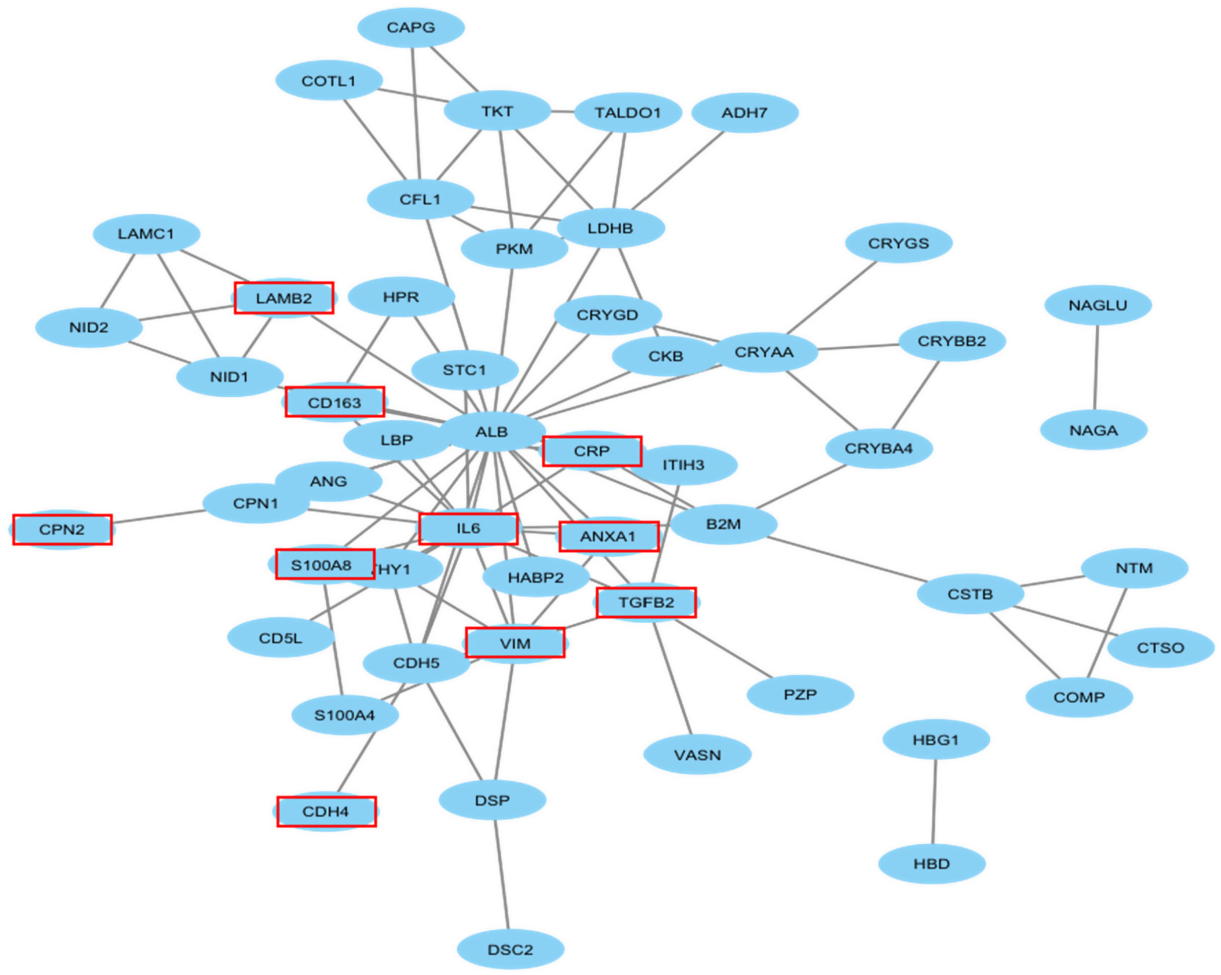
Protein–protein interaction (PPI) network of all DEPs. The PPI reflects the interactions among all significantly DEPs between the APAC group and control group. Proteins marked with red rectangles are associated with the PACG based on their biological function.

The PPI map displays interactions between proteins (Figure 6). From the interaction network, we identified multiple inflammatory factors, such as annexinA1 (ANXA1), interleukin‐6 (IL‐6), C‐reactive protein (CRP) and TGF‐β2. These factors influence each other and regulate downstream proteins, such as cadherin4 (CDH4), desmoplakin (DSP) and laminin subunit beta‐2 (LAMB2).

### 3.4 Verification of DEPs by ELISA

**FIGURE 7:**
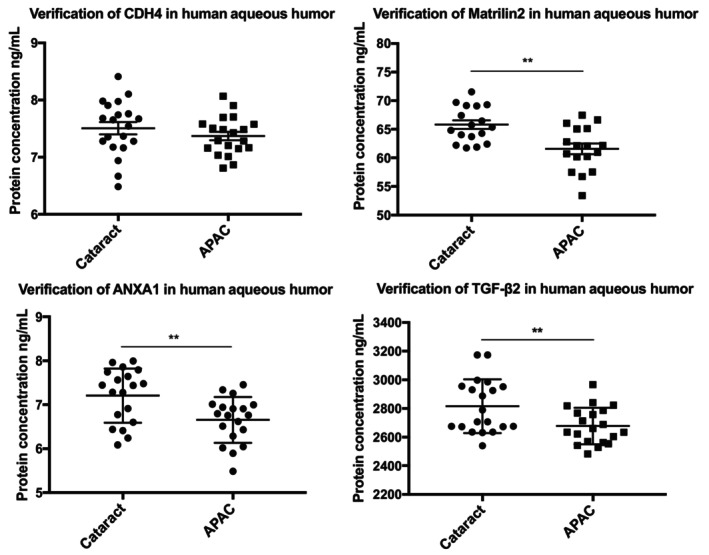
Verification of DEPs by ELISA. The MATN2, ANXA1 and TGF‐β2 in the aqueous humour (AH) are downregulated in the APAC group compared to the control. The expression of CDH4 in the AH is not significantly difference between the APAC and control group.

To further verify protein expression in the AH, samples from patients with APAC and cataract and patients with cataract only were collected again with the same standards as before in proteomic experiment. Patient information is summarized in Table 2. TGF‐β2, MATN2 and ANXA1 are downregulated in the APAC group compared to the control. The CDH4 does not show statistical difference between the groups. The trend of TGF‐β2 and MATN2 in the APAC group is consistent with the result in proteomic experiment. However, in comparison with control group, the ANXA1 in APAC group upregulate in the proteomic data and downregulate in the ELISA test (Figure 7).

## 4 DISCUSSION

APAC can obstruct the outflow of AH, causing higher IOP, which can lead to irreversible optic neuropathy and blindness.^28^ Previous research usually focused on neuroprotection less noticing the microenvironment of anterior chamber. AH not only provides nutrition support but also reflects the state of anterior chamber angle. Our research aimed to explore the differential proteins in the AH between the APAC group and the control group via proteomic technology and ELISA test and to seek proteins with underlying therapy value for APCG in future.

We measured the amount of protein in every one sample by the way of Bradford and found the concentration of protein was too little to carry out the proteomic test in control group. For solving the problem, we pooled 10 samples as a group for further test. We also analysed the reasons why protein concentration in the control group is lower than the APAC group. First, while collecting the samples, the condition of anterior chamber still be in an inflammatory state, the permeability of vessel was increased, more proteins flowed into the AH. Second, the iris and ciliary body secreted directly more proteins into anterior chamber in APAC patients. Third, the closure of anterior chamber angle slowed down the flow‐out of proteins in AH of the APAC.

More than 400 proteins were measured by proteome test, there were 91DEPs in comparison with control. In the 91 DEPs, though some proteins have been reported in glaucoma, like TGF‐β2, however, it is fewer about the report of the change in AH of early stage of PACG. The anterior chamber angle closure starts from a narrow angle to 360‐degree peripheral iris synechia accompanied with inflammatory reaction in the AH, ECM deposition in the trabecular meshwork, tissue adhesion and fibrous union in the anterior chamber angle.^29^ Based on the protein function, involved signalling pathways and ever reported glaucoma pathologic mechanisms, we chose some interesting proteins (marked yellow in Table 3). Besides, one sample only be enough for verification of four proteins in ELISA test. Among of those interesting proteins, four proteins were chosen, namely Matrilin2, AnnexinA1, TGF‐β2 and CDH4. Summarized from the uniport database and previous reported studies, the four proteins involved in matrix assembly or innate and adaptive immune response or cell adhesion or retinal development and so on. Therefore, their biological process is related with pathology of anterior chamber angle closure. We again measured their expression in AH via ELISA test with multiple independent samples.

The result of ELISA shows that the TGF‐β2 and MATN2 express at a lower level in the APAC group than in the control group. This result is consistent with data of the proteome. However, the expression of ANXA1 and CDH4 in ELISA is inconsistent with the proteome result. ANXA1 is upregulated in the APAC group compared with the control group in the proteomic experiment, whereas it is downregulated in the ELISA test. The CDH4 is downregulated in the APAC group compared with the control group in the proteomic experiment, whereas it is not significantly different in the ELISA.

The MATN2 is downregulated in the APAC group in both methods. MATN2 is widely distributed in many human tissues or organs, like the bone, heart, nerve and eye.^30^, ^31^, ^32^ The Uniprot database shows the MATN2 is involved in the ECM composition and acts as an adaptor protein in the ECM. MATN2 also paly roles in inflammation reactions, extracellular filamentous network formation and neurite growth. In terms of inflammation, one study showed that soluble MATN2 promotes inflammatory activity via activation of the TLR/PKR/NFKB signalling pathway in calcific aortic valve disease.^33^ Another study also revealed that elevated MATN2 induced inflammatory responses in post‐burn patient serum and the mechanism might be partly mediated by TLR4 in animal experiments.^34^ MATN2 can activate innate immune cells and could be involved in early axon injury in inflammatory diseases of the central nervous system.^35^ Moreover, MATN2 is also a poly‐adhesive connector protein, which cooperates with other proteins to participate in nerve growth and migration, as well as neuromuscular connection.^30^, ^36^ In our data, the expression of MATN2 was lower in the AH of the APAC group than in controls. However, levels of inflammatory factors, such as CRP, IL6 and CD163 are higher. This is contrary to the results of previous studies on the proinflammatory effect of MATN2. One reason for this difference might be that the level of MATN2 has been decreased after the attack and another reason is it does not act in acute response. The function of MATN2 in the glaucoma is unclear, especially in inflammation, ECM formation and nerve growth. Thus, the function of MATN2 is worthy of further research in the issues of anterior chamber angle, such as iris and trabecular meshwork.

The TGF‐β2 is downregulated based on the proteomic experiment and ELISA data. TGF‐β2 is an important factor in inflammation and fibrosis. Some signalling pathways including TGF‐β2 could modulate ECM formation in multiple organs, such as the eye, liver and lungs.^37^, ^38^, ^39^ Therefore, TGF‐β2 is used to design fibrosis models in vivo, including glaucoma and liver fibrosis.^40^, ^41^, ^42^ Studies have shown that TGF‐β2 could contribute to ECM deposition and inhibit ECM transformation in the AH or trabecular meshwork of glaucoma patients. The ECM deposition decreases drainage in the anterior chamber angle, increases IOP and exacerbates nerve damage.^43^, ^44^ Some researchers designed experiments to control the expression of TGF‐β2 to treat glaucoma.^45^, ^46^ In our research, the expression of TGF‐β2 was lower in the APAC group in comparison with controls.

The expression of ANXA1 and CDH4 was inconsistent in the two methods. Uniprot data reveal that ANXA1 is a vital anti‐inflammatory factor in humans. CDH4 is a type of calcium‐dependent cell adhesion protein, which preferentially interacts with itself in a homophilic manner in connecting cells. Cadherins could play an important role in retinal development. We suspect that the reason for this discrepancy might include the following: (1) the influence of IOP‐lowering drugs before surgery; (2) differences in the timing of sampling. However, the function of the two factors is also worth noting, especially the protein ANXA1.

Together, MATN2 and TGF‐β2 are consistently downregulated in the APAC group compared with controls in both methods. MATN2 and TGF‐β2 could interact with each other during biological processes. Comparing with previous data in some inflammation diseases, the MATN2/TGF‐β2 in this study does not show obvious increase in the AH of the APAC. However, Chen et al.^47^ found that the concentrations of TGF‐β1 and TGF‐β2 were significantly higher in AH samples from patients with APAC versus cataract. Guo et al.^48^ found that the concentration of TGF‐β2 in AH of primary open‐angle glaucoma patients, but not chronic angle‐closure glaucoma, primary angle‐closure suspect or acute angle‐closure glaucoma patients, was significantly higher than control eyes. However, the TGF‐β2 of acute angle‐closure glaucoma patients with high IOP (>21 mmHg) was significantly higher than those with normal IOP. Proteins in AH are changeable. Many factors affect proteins in AH, such as disease degree, circumstances, medicines and so on. Therefore, the TGF‐β2 maybe related with stage of the PACG. We have not found the report about the MATN2 in the AH.

We also summarize other studies on PACG based on the proteome. These studies were concentrated in Asia. They concluded that DEPs in the AH of PACG patients are mainly associated with the inflammation response, ECM aberration, optic nerve damage, lipid metabolism and cell death (Table 4). We identified the same proteins, including S100A8, CDH, TGF‐β2 and ANXA1. Therefore, it is necessary to seek suitable anti‐inflammatory, anti‐fibrotic and neuroprotective factors that will facilitate effective therapy for early glaucoma.

**TABLE 4:** Summary of proteomics studies of PACG.

PMID	Nation	Disease	Method	Verification method	Sample type	Sample size	Conclusions	Proteins
34240827	Saudi (PACG) Control (USA)	PACG	Orbitrap Q Exactive Plus mass spectrometer	Triple quadrupole mass spectrometer	Iris	PACG:Control = 48:5	426 upregulated proteins, 251 downregulated proteins, Titin (↑) and Obscurin (↓), Conclusion: extracellular matrices increased in PACG patients	ECM: brillar collagens, EMILIN‐2, fibrinogen, fibronectin, matrilin‐2; matricellular proteins: hrombospondin‐1; cell‐matrix interactions: integrins, laminin, histidine‐rich glycoprotein, paxillin; modulate ECM turnover: α‐2macroglobulin, tissue factor pathway inhibitor2, papilin
29536409	Singapore	PACG	Label‐free method	No	AH	PACG:Cataract = 2:3	501 upregulated proteins, 272 downregulated proteins. Conclusion: atypical collagens and fibronectins critical mediators of oxygen homeostasis and neuronal function	Downregulated proteins:TRMT61A, N4BP3, TMPRSS6, SLC4A7, NEDD4, CCT8, HNRNPU, GPR158, SLC25A31, EIF2AK4, MARCH5, GINS4, FABP12; Upregulated proteins: ACTB, PCDHGA2, CCNE2, PLTP, ASAH1, S100A8, F5, GTF2IRD2, CHSY1, KERA, LAMP2, DMKN, GSGIL, CRYZL1, C1RL, GABRE, C2, PATL1, CIQA, LYZ, SHANK1, SLC25A3, CST7, MRE11A, TRMT1L, TBX4, AP3M2, VDAC1
34926655	Beijing, China	AACG	Data‐independent acquisition (DIA)	Parallel reaction monitoring (PRM)	AH	Early AACG:late AACG = 25:27	87 proteins, Conclusion: prostaglandin‐H2 D‐isomerase was found to have the potential to evaluate optic nerve damage	Upregulated proteins: prostaglandin‐H2 D‐isomerase, fibrillin‐1, pigment epithelium‐derived factor, semaphorin‐7A, chitinase‐3‐like protein 1, cadherin‐2, decorin, fatty acid‐binding protein 5, semaphorin‐3B, cystatin‐M, haemoglobin subunit alpha, antileukoproteinase, cocaine and AMPHETAMINE‐regulated transcript protein, beta‐2 microglobulin. Downregulated proteins: complement factor H, alpha‐2‐HS‐glycoprotein vitronectin, fibrinogen gamma chain
29332228	India	PACG	Hybrid Orbitrap Velos Pro mass spectrometer	No	AH	PACG:Cataract = 9:9	Conclusion: vascular remodelling, blood coagulation and immune response pathways are majorly affected by glaucoma	Immune system processes: (↑) CD14, CD59, CFD, RIRREL3, APOA4, CHGA and MYB; Blood coagulation/regulation of body fluids (↑): TIMP1, CFD, CD59 and MYB; Response to light stimulus: (↑) MFAP4, AGRN, APOA4 and APOC3; Photo‐transduction and retinoic metabolic process: (↑) APOA4, AGRN and C3
34214668	China	PAACG	DIA (PAACG: Cataract = 33:23)	PRM (PAACG: Cataract = 24:10)	AH	PAACG:Cataract = 57:33	182 differentially expressed proteins in PAACG compared with cataract. Immune response, lipid metabolism and cell death; VTN, SERPIND1 and CD14 showed significant upregulation in glaucoma; PAACG was characterized with activation of inflammation response; SERPIND1 plays vital role in glaucoma occurrences	Differential proteins with higher levels in the glaucoma groups included apolipoproteins (APOA1, APOA2, APOA4, APOE, APOH), complement proteins (C1R, C2, C4A/C4B, C5, C6, C8A, C9, CFB, CFI) and inflammatory protein (SERPINA1, SERPINF2, CD14, GC, ITIH4). By contrast, proteins with lower expression levels included IGFBP4, IGFBP6, TGFB2 and ANXA1, which were mainly involved in cellular movement and development
30958601	India	PACG/NG‐Ctrls	AH (PACG/NG‐Ctrls = 8/8) TM (PACG/NG‐Ctrls = 3/3)	ELISA (*n* = 6)	AH and TM	AH (PACG:NG‐Ctrls = 8:8); TM (PACG:NG‐Ctrls = 3:3)	Fibulin‐7 was significantly low in AH of PACG in comparison with NG‐Ctrls. Ligatin has no statistical difference between the three groups	
30994369	India	PACG/Cataract	Liquid chromatography–mass spectrometry (*n* = 3)	Western blot and turbidimetric immunoassay	AH	PACG:Cataract = 72:78	In PACG: (↑) osteopontin (OPN), Cystatin C; (↓) cathepsin D. Elevated levels of OPN and cystatin C in PACG along with altered cathepsin levels may contribute to ECM aberration in glaucoma	Proteomics analysis identified 184 and 299 proteins in control and PACG

However, there are limitations to this study. One limitation is we pooled samples in each group of proteome test. This design might decrease the reliability of result. For enhancing the reliability, we again verified some proteins by ELISA test with multiple independent samples. Another is the samples originated from a patient undergoing surgery rather than from disease onset, and this preoperative treatment could have influenced protein expression. Besides, the number of samples was limited in the proteome and ELISA test, and thus, we will require additional samples in different stage of disease to obtain better data. Lastly, the eye drops before surgery may affect the proteins in the AH. In the future, we hope to generate a better animal model to investigate the specific functions and mechanisms of these factors in glaucoma.

## 5 CONCLUSION

In summary, this study analysed the proteome of AH in patients with APAC versus cataract only. It determined that the DEPs are related to inflammation, fibrosis and nerve growth. It was found that low level of MATN2/TGF‐β2 expressed in the AH of APAC. Although there is acute inflammation response in the AH of the APAC, the response differs with the PACG, which provide us an information that we need to take different anti‐inflammation measures in different stages of the PACG.

## AUTHOR CONTRIBUTIONS

**Liming Wang:** Conceptualization (equal); data curation (equal); formal analysis (equal); writing – original draft (equal); writing – review and editing (equal). **Zhao Xu:** Formal analysis (equal); supervision (equal); validation (equal); writing – original draft (equal). **Yaru Hong:** Data curation (equal); resources (equal); validation (equal). **Yan Liu:** Conceptualization (equal); resources (equal); supervision (equal). **Xiaomin Zhang:** Project administration (equal); resources (equal). **Qiang Feng:** Funding acquisition (equal); resources (equal); supervision (equal). **Dandan Zhang:** Formal analysis (equal); resources (equal). **Kexi Chen:** Funding acquisition (equal); resources (equal). **Guli Humaer Yiming:** Formal analysis (equal); resources (equal). **Xiaorong Li:** Conceptualization (equal); funding acquisition (equal); project administration (equal); resources (equal). **Aihua Liu:** Data curation (equal); funding acquisition (equal); project administration (equal); resources (equal). **Lijie Dong:** Methodology (equal); project administration (equal); supervision (equal); writing – review and editing (equal).

## FUNDING INFORMATION

This work was supported by a grant from General Project of Natural Science Foundation of Xinjiang Uygur Autonomous Region (No: 2020D01A06). This work was supported by grants from Tianjin Binhai New Area Health Commission Science and Technology Project (2022BWKZ003).Tianjin Health Research Project (TJWJ2023ZD002); Tianjin Higher Education Commission Science and Technology Development Fund Project (2022ZD057); Open Project of Tianjin Key Laboratory of Retinal Functions and Diseases (2021tjswmm002); The Science & Technology Development Fund of Tianjin Education Commission for Higher Education (2019ZD030); Tianjin Key Medical Discipline (Specialty) Construction Project (TJYXZDXK‐037A).

## CONFLICT OF INTEREST STATEMENT

The authors declare no conflicts of interest.

## Data Availability

The data that support the findings of this study are available from the corresponding author, [Lijie Dong], upon reasonable request.
